# Maternal immune system adaptation to pregnancy - a potential influence on the course of diabetic retinopathy

**DOI:** 10.1186/1477-7827-8-124

**Published:** 2010-10-21

**Authors:** Snježana Kaštelan, Martina Tomić, Josip Pavan, Slavko Orešković

**Affiliations:** 1 Department of Ophthalmology, Clinical Hospital "Dubrava", Avenija Gojka Šuška 6, 10000 Zagreb, Croatia; 2 Department of Diabetic Complications, Division for Ophthalmology, University Clinic for Diabetes, Endocrinology and Metabolic Diseases, Medical Faculty University of Zagreb, "Vuk Vrhovac" Institute, Dugi dol 4a, 10000 Zagreb, Croatia; 3 Department of Gynecology and Obstetrics, Zagreb University School of Medicine Petrova 13, 10000 Zagreb, Croatia

## Abstract

**Background:**

Progression of diabetic retinopathy occurs at least temporarily during pregnancy. Although the cause of this progression is not entirely understood, the immune phenomenon and chronic inflammation may play a significant role. During pregnancy in order to avoid fetus rejection, certain components of the immune system that are knowingly implicated in the pathogenesis of diabetic retinopathy are activated including generalized leukocyte activation and an increase in certain cytokine plasma levels. Activated leukocytes with up regulated adhesion molecules have an increased potential to bind to the endothelium cells of blood vessels. Leukocyte-endothelial interaction and the consequent leukostasis with capillary occlusion, ischemia and vascular leakage have a substantial role in the development of diabetic retinopathy. Furthermore, certain increased cytokines are known to cause blood-retinal-barrier breakdown whilst others promote angiogenic and fibrovascular proliferation and thereby can also be implicated in the pathogenesis of this diabetic complication.

**Presentation of the hypothesis:**

We hypothesized that the activation of the immune system during gestation may have an influence on the course of retinopathy in pregnant diabetic women.

**Testing the hypothesis:**

We suggest two prospective follow up studies conducted on women with type 1 diabetes mellitus. The first study would include a group of non-pregnant women and a group of diabetic women undergoing normal pregnancy matched for age and duration of diabetes. In the second study pregnant women would be divided into two groups: one with normal pregnancy and the other with preeclampsia. The procedure and data collection in both studies will be identical: a complete ophthalmological examination, glycaemic control, blood pressure measurement and venous blood samples for the determination of plasma levels of cytokines (TNF-alpha, IL-1beta, IL-6, IL-8) and adhesion molecules (ICAM-1, VCAM-1).

**Implications of the hypothesis:**

Considering the present assumption, the gestational immune activation could be suggested as a potential risk factor for the development and progression of retinopathy in diabetic women. A better understanding of immunomodulatory effects of pregnancy on diabetic retinopathy pave the way for further investigations of the mechanism of its pathogenesis and could be essential for novel approaches to the treatment of this serious sight threatening complication of diabetes mellitus.

## Background

Diabetic retinopathy is a common and progressive microvascular complication of diabetes mellitus and the leading cause of new blindness in the working age population in developed countries [1]. Numerous studies have formerly demonstrated a deterioration of retinopathy in diabetic women during pregnancy [2-4]. Although many researchers have succeeded to clarify the connection between retinopathy development and gestation, the exact mechanism responsible for this is still not entirely clear [2-9]. The consensus is that this mechanism is multifactorial with important contributory factors including hyperglycaemia [3,4,7,8], duration of diabetes before conception [3,4], baseline status of retinopathy [3,4,8], rapid control of blood glucose during pregnancy [3,8], coexisting hypertension [2], preeclampsia [9] and changes in retinal blood flow [9].

It has previously been established that immune phenomenon and inflammatory reactions are involved in the pathogenesis and progression of diabetic retinopathy [10-13]. During pregnancy the maternal circulating immune system undergoes modifications in cell counts, phenotypes as well as the function and ability to produce soluble factors such as cytokines. Different components of the immune system maintain a balance between the activation and suppression of the innate and adaptive immune system branches. This in turn allows the maternal defense mechanism capabilities to remain intact and simultaneously plays a central role in the maternal adaptation in pregnancy to avoid fetal allograft rejection [14].

Increased level of hormones (estrogen, progesterone and prolactin) during pregnancy may also contribute to the changes of the maternal immune system. Immune and endocrine cells possess the ability to synthesize and express receptors for cytokines and hormones and by binding to these receptors they can modulate the activity of immune and endocrine cells. Thus the relationship of these two systems and their mutual interactions via cytokines which act as mediators enable hormones to also have an effect on the course of diabetic retinopathy during pregnancy [15].

A recent study addressing the association of inflammatory markers and diabetic retinopathy in pregnancy failed to find statistically significant increases in levels of interleukin-6 (IL-6) and vascular adhesion molecule-1 (VCAM-1) in the plasma of diabetic women compared to non-diabetic controls, although the IL-6 value tended to be higher in diabetic as opposed to nondiabetic women [5]. However, this investigation did not draw a comparison between obtained values and the values of these same parameters in non-pregnant diabetic women. In our opinion this problem should be considered from another point of view. To clarify this issue it is essential that the level of certain cytokines and adhesion molecules as well as IL-6 and VCAM-1 is raised in pregnant compared to non-pregnant women as a result of adaptation of the immune system during pregnancy. In previous studies it has already been confirmed and proven that all pregnant women demonstrate increased levels of IL-6, IL-8, adhesion molecules and activation of leukocytes in relation to the condition prior to pregnancy [14,16-19]. We therefore presume that in healthy women during gestation this increase does not cause pathological changes whilst in pregnant diabetic patients especially those with previous retinopathy and longer duration of diabetes it may cause a progression of this complication. In fact, increased levels of these molecules which are found in all women, with and without diabetes, caused by pregnancy could therefore influence retinopathy progression in diabetic women. However, we maintain that the differences between pregnant and non pregnant women are essential and not the differences between diabetic and non diabetic patients alone. Hence, one could speculate that pregnancy itself may be one of the causing factors for retinopathy progression during gestation.

Thus, considering the existing knowledge and our own presumption, it is reasonable to assume that the adaptation of the immune system during gestation may also contribute and be one of the causing factors for retinopathy progression in pregnant diabetic women.

### Presentation of the hypothesis - how the maternal immune system could influence the progression of diabetic retinopathy during pregnancy

**Figure 1:**
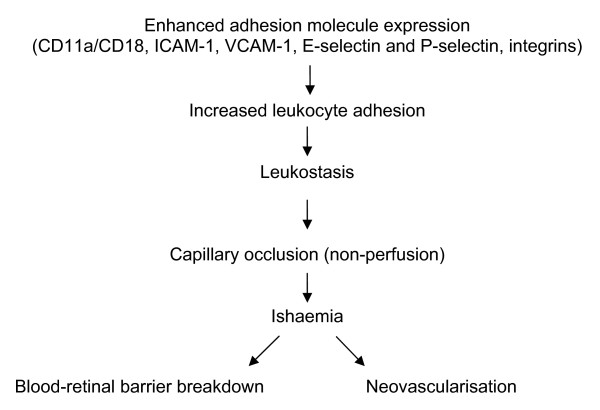
**Pathogenesis of diabetic retinopathy**. Abbreviations: ICAM, intercellular adhesion molecule; VCAM, vascular cell adhesion molecule.

Several studies have been conducted in order to explain the mechanisms underlying the progression of diabetic retinopathy during pregnancy [2-9]. After adjusting to the duration of diabetes, preexisting retinopathy prior to conception, glycaemic and blood pressure control, current pregnancy was found to be an important risk factor in retinopathy progression. However, the exact mechanism by which this progression occurs is not entirely clear [2-9]. Capillary occlusion represents a characteristic pathologic feature in early diabetic retinopathy and is presumed to cause capillary non-perfusion, endothelial cell damage and initiate a blood-retinal barrier breakdown as well as neovascularisation [20] [Figure 1]. The exact mechanism by which capillary occlusion occurs is still unclear but extending evidence suggests that it is associated with increased leukocyte adhesion to the diabetic retinal vasculature. The increased leukocyte-endothelial cell adhesion and consecutive leuokostasis is a result of enhanced adhesion molecule expression such as CD11a/CD18, intercellular adhesion molecule-1 (ICAM-1), VCAM-1, E-selectin and P-selectin on endothelial cells and leukocytes as well as up regulation of integrins which represent their counter-receptors [21]. Recent reports have described normal pregnancy as characterized by an activation of circulating leukocytes as part of a generalized immune response. This leukocyte activation is marked by up-regulation in the expression of different adhesion molecules such as CD11a,b/CD18, CD54 (ICAM-1) and CD49d as well as integrins and selectins present on the surface of circulating leukocytes. Furthermore these activated leukocytes have the increased potential to bind to the endothelium of blood vessels via interactions with ICAM-1 and VCAM-1 molecules [16-18]. This demonstrates that one of the essential pathologic events in the development of diabetic retinopathy is activated during pregnancy and could therefore be at least partially responsible for its progression in the gestation period.

Several investigations have shown an increase in concentration of cytokines, growth factors and adhesion molecules in patients with diabetic retinopathy which may consequently stimulate endothelial cells and trigger neovascularisation [Table 1]. In patients with proliferative diabetic retinopathy increased vitreous concentrations of the interleukin-1beta (IL-1beta), IL-6, soluble IL-2 receptor (sIL-2R) and IL-8 were found [12], whilst the serum of same patients contained elevated levels of tumor necrosis factor-alpha (TNF-alpha), IL-6, IL-8 and sIL-2R [12,13,22]. Furthermore, it is also well known that some cytokines are potent vascular permeability factors. They may contribute to the early retinal microvascular abnormalities induced by diabetes as well as capillary leakage which is responsible for hard exudates formation and macular edema development. Accumulating evidence suggests that different cytokines also play major roles both in the establishment and in the maintenance of normal human pregnancy [18,19,23]. It is documented that the serum value of IL-1beta, IL-6 [19] and IL-8 [16] increase during the gestation period. During pregnancy IL-6 in the serum of healthy women progressively increased, reaching the highest levels in the third trimester with a further increment at the onset of labour [24]. It is well documented that IL-1beta up-regulates adhesion molecules which are in turn vital for the binding of leukocytes to the endothelial surface [25], whilst IL-6 and IL-8 are among the most essential cytokines implicated in the development of diabetic retinopathy [12,13]. IL-6 and IL-8 are pro-inflammatory cytokines that cause blood-retinal barrier breakdown by opening tight junctions between retinal vascular endothelial as well as the pigmented epithelial cells. They therefore promote increased vascular permeability and leakage and thus participate in the pathogenesis of diabetic retinopathy [13]. IL-8 has an angiogenic and fibrovascular proliferative effect on ocular tissue and is implicated in processes of neovascularisation and thereby in the development of proliferative diabetic retinopathy [13,26]. The above mentioned activation of these immunological mediators strongly suggests that they take part in the development of diabetic retinopathy during the gestation period.

**Table 1:** Cytokines, growth factors and adhesion molecules involved in diabetic retinopathy*

Parameter	DM without DR	DM with NPDR	DM with PDR
TNF-α	No change	Increased	Increased
IL-1α	Reduced	Reduced	Increased
IL-1β	No change	Increased	No change
IL-4	No change	Increased	Reduced
IL-6	No change	Increased	Increased
IL-8	Increased	Increased	Increased
IL-10	Reduced	No change	Increased
VEGF	Increased	Increased	Increased
EGF	Increased	Increased	Increased
ICAM-1	Increased	Increased	Increased
VCAM-1	Increased	Increased	Increased
E-selectin	Increased	Increased	Increased

* Changes relative to levels in non-diabetic control subjects

Abbreviations: DM, diabetes mellitus; DR, diabetic retinopathy; NPDR, nonproliferative diabetic retinopathy; PDR, proliferative diabetic retinopathy; TNF, tumor necrosis factor; IL, interleukin; VEGF, vascular endothelial growth factor; EGF, epidermal growth factor; ICAM, intercellular adhesion molecule; VCAM, vascular cell adhesion molecule

Preeclampsia is a well recognized risk factor for the progression and deterioration of diabetic retinopathy during pregnancy [9]. It is associated with increased systemic vascular resistance, enhanced platelet aggregation, activation of the coagulation system and endothelial cell dysfunction as part of a general inflammatory reaction [27,28]. A natural inflammatory response whilst less prominent is also a feature of healthy pregnancy in the third trimester [16,17]. In actual fact the results of several studies show that the progression of retinopathy was particularly observed at this time [4,8,29]. According to some investigations, a generalized inflammatory response in preeclampsia [27,28] is significantly increased when compared to normal pregnant and non-pregnant control subjects [30] [Table 2]. It is noteworthy that both TNF-α and IL-6 are well recognized as potential mediators of endothelial dysfunction in preeclampsia. IL-1β and IL-8 are able to induce endothelial cell activation and upregulation of endothelial adhesion molecules, which mediate the attachment of circulating monocytes to the endothelium [27,28,31,32]. This observation supports and explains the concept that pre-eclampsia is one of the risk factors for diabetic retinopathy development during pregnancy.

**Table 2:** Cytokines and adhesion molecules in the third trimester of normal pregnancy and pre-eclampsia*

Parameter	Normal pregnancy	Pre-eclampsia
TNF-α	Increased	Increased
IL-1α	No change	Increased
IL-1β	Increased	Increased
IL-4	No change/reduced	No change/reduced
IL-6	Increased	Increased
IL-8	Increased	Increased
IL-10	No change/increased	No change/increased
ICAM-1	Increased	Increased
VCAM-1	Increased	Increased

* Changes relative to levels in non-pregnant control subjects

Abbreviations: DM, diabetes mellitus; DR, diabetic retinopathy; NPDR, nonproliferative diabetic retinopathy; PDR, proliferative diabetic retinopathy; TNF, tumor necrosis factor; IL, interleukin; ICAM, intercellular adhesion molecule; VCAM, vascular cell adhesion molecule

It is commonly believed that the severity of diabetic retinopathy may regress at least to some degree in the postpartum period, although the rate and timing of this regression is still unknown [2,29,33]. The diabetic women should therefore be closely monitored until retinopathy is stabilized [33]. It is assumed that the impact and the immunological influence of pregnancy remains till one year after delivery and that these postpartal changes may be related to the course of some autoimmune diseases during this period [34].

It is a well-established fact that diabetes takes longer than five years to develop visible morphological changes corresponding to diabetic retinopathy [20,35]. Women with gestational diabetes certainly would not develop diabetic retinopathy during pregnancy and therefore fundus examination in these women is not required despite of their glucose blood level. In these women hyperglycemia and all its possible consequences do not have enough time to develop any complications resulting from diabetes [36]. Increased levels of certain factors involved in the development of diabetic retinopathy (such as cytokines and adhesion molecules as well as leukocyte activation) during nine months of pregnancy in healthy women do not cause alterations in the function and structure of blood vessels and therefore logically could not lead to microangiopathy. Therefore, in healthy women changes accompanying the adaptation of the immune system to pregnancy remain without consequences. Alternatively, in women with diabetes changes in the immune system and subsequent increased levels of certain well-known risk factors throughout a period of nine months may lead to a deterioration of retinopathy. This is therefore consistent with established data that the increased values of these risk factors are connected to the progression of this complication in all diabetic patients [20,36]. Indeed, this is further supported by clinical observations which prove that the duration of disease and degree of retinopathy prior to conception are established risk factors for its progression during pregnancy [3,4,8].

### Testing the hypothesis

Since our intention is to explore the impact of some immunological changes during pregnancy on the development of diabetic retinopathy we need to compare a group of pregnant diabetic women and non-pregnant diabetic women who are matched for age and duration of the diabetes. With this knowledge we could therefore establish whether adaptation of the immune system during pregnancy in women with diabetes causes an increase in the level of certain risk factors which could additionally lead to an acceleration of retinopathy.

To verify our hypothesis we suggest two prospective follow up studies conducted on women with type 1 diabetes mellitus. The first study should contain a group of pregnant diabetic women undergoing normal pregnancy and a group of non-pregnant diabetic women matched for age and duration of diabetes. The second investigation should include pregnant diabetic women divided in two groups: a group with normal pregnancy and a group with complicated pregnancy namely preeclampsia. The procedure and data collection in both investigations will be identical.

Pregnant diabetic women should be recruited as soon as their pregnancy is diagnosed (usually between 5 and 10 weeks of gestation). They will be studied at the 12^th ^- 14^th ^week, 24^th ^- 26^th ^and 34^th ^- 36^th ^week of the gestation, and at 1 month, at 2 - 4 months, 6 - 7 months and 11 - 12 months postpartum. In the non-pregnant diabetic participants venous blood samples will be taken and regular check ups will be conducted every 3 months during an 18 month follow up period. Thus every visit will comprise of a specified set of procedures. They will include: a complete ophthalmological examination, glycaemic control, blood pressure measurement and venous blood samples for the determination of plasma levels of cytokines (TNF-α, IL-1β, IL-6, IL-8) and adhesion molecules (ICAM-1, VCAM-1). The pregnant women will also give urine samples in order to determine the presence and level of urine proteins.

A complete ophthalmological examination will include measurement of visual acuity, intraocular pressure measurement with Goldmann applanation tonometer, biomicroscopy, indirect ophthalmoscopy and fundus photography. Diabetic retinopathy will be graded from fundus photographs which will be taken through dilated pupils (by the use of tropicamide 5 mg/ml). The severity of retinopathy will be assessed using two 50° color slides, one centered at the macula and the other at the optic nerve head disc and will be graded using a modification of the Early Treatment Diabetic Retinopathy Study (ETDRS) grading system [37].

The progression of retinopathy will be classified as follows:

(1) No progression: no change or a decrease in DCCT score during pregnancy, and

(2) Progression: an increase in the DCCT score by one or more levels during pregnancy.

Glycaemic control will be assessed by measuring the level of glycosylated haemoglobin (HbA_1_c). Blood pressure will be regarded as increased when the following two criteria are met at two measuring sessions of at least 24 h apart: first, diastolic pressure is increased 15 mmHg or more from the first measurement during pregnancy until the end of pregnancy and secondly, when the final level has reached 90 mmHg or more. Chronic hypertension and pregnancy-induced hypertension are defined as high blood pressure before 20 weeks and after 20 weeks of gestation respectively.

Preeclampsia is defined as high blood pressure and proteinuria (0.3 g or more/24 h) after 20 weeks of gestation.

### Implications of the hypothesis

In relation to previous data of known mechanisms of diabetic retinopathy development [20-22,26] and the established fact that some of the same immunological changes occur during pregnancy [14,16-19,23,24], it is therefore reasonable to hypothesis that activation of the immune system during pregnancy could indeed participate in the aggravation of the course of retinopathy in diabetic women.

It is possible that in diabetic women this could originate or accelerate biochemical and molecular mechanisms and pathways contributing to blood-retinal barrier disruption, capillary occlusion and neovascularisation. These assumptions also help explain how pregnancy could participate in the development and progression of retinopathy in diabetic women even in the presence of good metabolic control and when retinopathy prior to conception is minimal.

Knowledge of the actual pathogenetic mechanism of diabetic retinopathy progression during pregnancy may deem important to the fields of diabetology, endocrinology and ophthalmology. Understanding the risk factors contributing to the aggravation of diabetic retinopathy during this period is helpful in designing criteria for the team management of pregnant women with diabetes.

We should bear in mind that after pregnancy in a certain number of diabetic women pathological fundus changes could regress at least in part [2,29]. Thus it would be interesting to investigate and clarify the immunological and other changes and conditions that occur during this period with the aim of explaining the pathogenesis of this regression. The understanding of the natural mechanisms that initiate regression of retinopathy would be particularly significant and more beneficial than understanding of the pathogenesis of retinopathy formation alone. All this acquired knowledge would be constructive and valuable in the development and improvement of therapy related to this complication of diabetes which in turn could then imitate the natural physiological conditions and mechanisms that cause regression after birth.

The above mentioned observations pave the way for further investigations of the mechanism of pathogenesis of diabetic retinopathy development and even possible regression. Moreover, knowledge of the molecules and pathways involved may create new therapeutic options. Needless to say, future research would clarify this issue. A better understanding of immunomodulatory effects of pregnancy on diabetic retinopathy would be essential for novel approaches to the treatment of this serious sight threatening complication of diabetes mellitus.

## Competing interests

The authors declare that they have no competing interests.

## Authors' contributions

All authors through continual scientific consultation have substantially participated and contributed in the conception and elaboration of the presented hypotheses. SK conceived the basic idea and wrote the manuscript. MT helped to draft the manuscript. All authors read and approved the final manuscript.

## References

[B1] Moss S Klein R Klein B The 14-year incidence of visual loss in a diabetic population Ophthalomology 1998 105 Suppl 6 998 1003 10.1016/S0161-6420(98)96025-0 9627648

[B2] The Diabetes Control and Complications Trial Research Group Effect of pregnancy on microvascular complications in the diabetes control and complications trial. The Diabetes Control and Complications Trial Research Group Diabetes Care 2000 23 1084 1091 10.2337/diacare.23.8.1084 10937502 PMC2631985

[B3] Klein BEK Moss SE Klein R Effect of pregnancy on progression of diabetic retinopathy Diabetes Care 1990 13 Suppl 1 34 40 10.2337/diacare.13.1.34 2404715

[B4] Rahman W Rahman FZ Yassin S Al-Suleiman SA Rahman J Progression of retinopathy during pregnancy in type I diabetes mellitus Clin Experiment Ophthalmol 2007 35 10 231 236 17430509 10.1111/j.1442-9071.2006.01413.x

[B5] Loukovaara S Immonen I Koistinen R Hiilesmaa V Kaaja R Inflammatory markers and retinopathy in pregnancies complicated with type I diabetes Eye 2005 19 4 422 430 10.1038/sj.eye.6701499 15286667

[B6] Kastelan S Tomić M Mrazovac V Kastelan Z Does maternal immune system alternation during pregnancy influence the progression of retinopathy in diabetic women? Med Hypotheses 2008 71 3 464 465 10.1016/j.mehy.2008.04.003 18479838

[B7] Phelps RI Sakol P Metzger BE Jampol LM Freinkel N Changes in diabetic retinopathy during pregnancy: correlations with regulation of hyperglycemia Arch Ophthalmol 1986 104 1806 1810 3789980 10.1001/archopht.1986.01050240080044

[B8] Chew EY Mills JL Metzger BE Remaley NA Jovanovic-Peterson L Knopp RH Conley M Rand L Simpson JL Holmes LB Metabolic control and the progression of retinopathy. The diabetes in early pregnancy study Diabetes Care 1995 18 631 637 10.2337/diacare.18.5.631 8586000

[B9] Kaaja R Loukovaara S Progression of retinopathy in type 1 diabetic women during pregnancy Curr Diabetes Rev 2007 3 Suppl 2 85 93 10.2174/157339907780598252 18220659

[B10] Baudouin C Fredj-Reygrobellet D Brignole F Lapalus P Gastaud P MHC class II antigen expression by ocular cells in proliferative diabetic retinopathy Fundam Clin Pharmacol 1993 7 Suppl 9 523 530 10.1111/j.1472-8206.1993.tb00256.x 8314197

[B11] Tang S Le-Ruppert KC Activated T lymphocytes in epiretinal membranes from eyes of patients with proliferative diabetic retinopathy Graefes Arch Clin Exp Ophthalmol 1995 233 11 21 25 10.1007/BF00177781 7721119

[B12] Yuuki T Kanda T Kimura Y Kotajima N Tamura J Kobayashi I Kishi S Inflammatory cytokines in vitreous fluid and serum of patients with diabetic vitreoretinopathy J Diabetes Complications 2001 15 5 257 259 10.1016/S1056-8727(01)00155-6 11522500

[B13] Doganay S Evereklioglu C Er H Türköz Y Sevinç A Mehmet N Savli H Comparison of serum NO, TNF-α, IL-1β, sIL-2R, IL-6 and IL-8 levels with grades of retinopathy in patients with diabetes mellitus Eye 2002 16 2 163 170 10.1038/sj/eye/6700095 11988817

[B14] Luppi P How immune mechanisms are affected by pregnancy Vaccine 2003 21 24 3352 3357 10.1016/S0264-410X(03)00331-1 12850338

[B15] Zen M Ghirardello A Iaccarino L Tonon M Campana C Arienti S Rampudda M Canova M Doria A Hormones, immune response, and pregnancy in healthy women and SLE patients Swiss Med Wkly 2010 140 13-14 187 201 20175004 10.4414/smw.2010.12597

[B16] Luppi P Haluszczak C Betters D Richard CA Trucco M DeLoia JA Monocytes are progressively activated in the circulation of pregnant women J Leukoc Biol 2002 72 5 874 884 12429709

[B17] Luppi P Haluszczak C Trucco M Deloia JA Normal pregnancy is associated with peripheral leukocyte activation Am J Reprod Immunol 2002 47 2 72 81 10.1034/j.1600-0897.2002.1o041.x 11900591

[B18] Wegmann TG The cytokine basis for the cross talk between the maternal immune and reproductive systems Curr Opin Immunol 1990 2 5 765 769 10.1016/0952-7915(90)90048-L 2701982

[B19] Austgulen R Lien E Liabakk NB Jacobsen G Arntzen KJ Increased levels of cytokines and cytokine activity modifiers in normal pregnancy Eur J Obstet Gynecol Reprod Biol 1994 57 3 149 155 10.1016/0028-2243(94)90291-7 7713287

[B20] Davis MD Diabetic retinopathy. A clinical overview Diabetes Care 1992 15 12 1844 1874 10.2337/diacare.15.12.1844 1464242

[B21] Chibber R Ben-Mahmud BM Chibber S Kohner EM Leukocytes in diabetic retinopathy Curr Diabetes Rev 2007 3 1 3 14 10.2174/157339907779802139 18220651

[B22] Mitamura Y Harada C Harada T Role of cytokines and trophic factors in the pathogenesis of diabetic retinopathy Curr Diabetes Rev 2005 1 1 73 81 10.2174/1573399052952596 18220584

[B23] Amoudruz P Minang JT Sundström Y Nilsson C Lilja G Troye-Blomberg M Sverremark-Ekström E Pregnancy, but not allergic status, influences spontaneous and induced interleukin-1β (IL-1β), IL-6, IL-10 and IL-12 responses Immunology 2006 119 1 18 26 10.1111/j.1365-2567.2006.02400.x 16764689 PMC1782335

[B24] Opsjln SL Wathen NC Tingulstad S Wiedswang G Sundan A Waage A Austgulen R Tumor necrosis factor, interleukin-1, and interleukin-6 in normal human pregnancy Am J Obstet Gynecol 1993 169 1 397 404 8362955 10.1016/0002-9378(93)90096-2

[B25] Meager A Cytokine regulation of cellular adhesion molecule expression in inflammation Cytokine Growth Factor Rev 1999 10 1 27 39 10.1016/S1359-6101(98)00024-0 10379910

[B26] Simo R Carrasco E Garcia-Ramirez M Hernandez C Angiogenic and antiangiogenic factors in proliferative diabetic retinopathy Curr Diabetes Rev 2006 2 1 71 98 10.2174/157339906775473671 18220619

[B27] Dekker GA Sibai BM Etiology and pathogenesis of preeclampsia: current concepts Am J Obstet Gynecol 1998 179 1359 1375 10.1016/S0002-9378(98)70160-7 9822529

[B28] Redman CW Sacks GP Sargent IL Preeclampsia: an excessive maternal inflammatory response to pregnancy Am J Obstet Gynecol 1999 180 499 506 10.1016/S0002-9378(99)70239-5 9988826

[B29] Ohrt V The influence of pregnancy on diabetic retinopathy with special regard to the reversible changes shown in 100 pregnancies Acta Ophthalmol 1984 62 603 616 10.1111/j.1755-3768.1984.tb03973.x 6485756

[B30] Luppi P Deloia J Moncytes of preeclamptic women spontaneously synthesize pro-inflammatory cytokines Clin Immun 2006 118 268 275 10.1016/j.clim.2005.11.001 16337193

[B31] Kauma S Takacs P Scordalakes C Walsh S Green K Peng T Increased endothelial monocyte chemoattractant protein-1 and interleukin-8 in preeclampsia Obstet Gynecol 2002 100 706 714 10.1016/S0029-7844(02)02169-5 12383538

[B32] Leik CE Walsh SW Neutrophils infiltrate resistance-sized vessels of subcutaneous fat in women with preeclampsia Hypertension 2004 44 72 77 10.1161/01.HYP.0000130483.83154.37 15148293

[B33] Chan WC Lim LT Quinn MJ Knox FA McCance D Best RM Management and outcome of sight-threatening diabetic retinopathy in pregnancy Eye 2004 18 826 832 10.1038/sj.eye.6701340 14976547

[B34] Watanabe M Iwatani Y Kaneda T Hidaka Y Mitsuda N Morimoto Y Amino N Changes in T, B, and NK lymphocyte subsets during and after normal pregnancy Am J Reprod Immunol 1997 37 5 368 377 9196795 10.1111/j.1600-0897.1997.tb00246.x

[B35] Borch-Johnsen K Epidemiology of microangiopathy in type 1 diabetes mellitus. A review Diabetes Metab 1993 19 133 137 8314416

[B36] Bloomgarden ZT Screening for and managing diabetic retinopathy: current approaches Am J Health Syst Phar 2007 64 Suppl 12 S8 14 10.2146/ajhp070331 17720893

[B37] Early Treatment Diabetic Retinopathy Study Research Group Grading diabetic retinopathy from stereoscopic colour fundus photographs: an extension of the modified Arlie House classification. ETDRS Report Number 10 Ophthalmology 1991 98 986 806 2062513

